# Synthesis of 2,6-disubstituted tetrahydroazulene derivatives

**DOI:** 10.3762/bjoc.8.77

**Published:** 2012-05-04

**Authors:** Zakir Hussain, Henning Hopf, Khurshid Ayub, S Holger Eichhorn

**Affiliations:** 1 Institut für Organische Chemie, Technische Universität Braunschweig, Hagenring 30, D-38106 Braunschweig, Germany; Fax: +49 5313915388; 2 Department of Chemistry, COMSATS Institute of Information Technology (CIIT), University Road, Abbottabad 22060, KPK, Pakistan; Fax: +92 (992) 383441; 3 Department of Chemistry and Biochemistry, University of Windsor, 401 Sunset Avenue, Essex Hall, Windsor, ON Canada N9B 3P4, Fax: +1 (519) 973-7064

**Keywords:** carbene addition, hydroazulenes, liquid crystals, ring expansion

## Abstract

Synthesis of hydroazulene derivatives has been carried out through a ring-enlargement route by using carbene adduct intermediates. The protocol can be applied for the construction of functionalized hydroazulene skeletons as key components of many natural products as well as the core system of novel liquid-crystalline materials.

## Introduction

Hydroazulene skeletons provide the basic ring systems of natural products, such as guaianolide sesquiterpenes [1–2] and the so-called furanether B series [3–4]. The stereoselective synthesis of *trans*-hydroazulene derivatives by a tandem Michael/intramolecular Wittig approach was reported previously [5]. Recently, a new synthetic method for the construction of a hydroazulene skeleton by a [5 + 2]cycloaddition reaction was also developed [6]. Additionally, there have been many reports on the synthesis of 2,6-disubstituted azulenes [7–8]. In 1951, the ring expansion of indanes was reported for the synthesis of such systems, but it delivered only very low yields [9]. We recently employed [10–11] the method reported by Keehn et al. [12] using Birch reduced indanes for the synthesis of 2,6-disubstituted perhydroazulene systems as core elements of novel liquid-crystalline (LC) materials.

As far as mesogenic compounds are concerned, there is still a growing need for more new derivatives to be synthesised and tested for features desirable for novel displays, which include, but are not limited to, lower driving voltages, lower power consumption and faster response times. In contrast to the core system presently used in most nematic LC materials, our new core based on perhydroazulenes showed [13], e.g., improved properties regarding phase-transition temperatures. Therefore, due to its potential use as a subsystem of many natural products, as well as as the core moiety for novel LC materials, further investigation on perhydroazulene-based substrates and synthetic intermediates is desirable.

## Results and Discussion

**Scheme 1:**
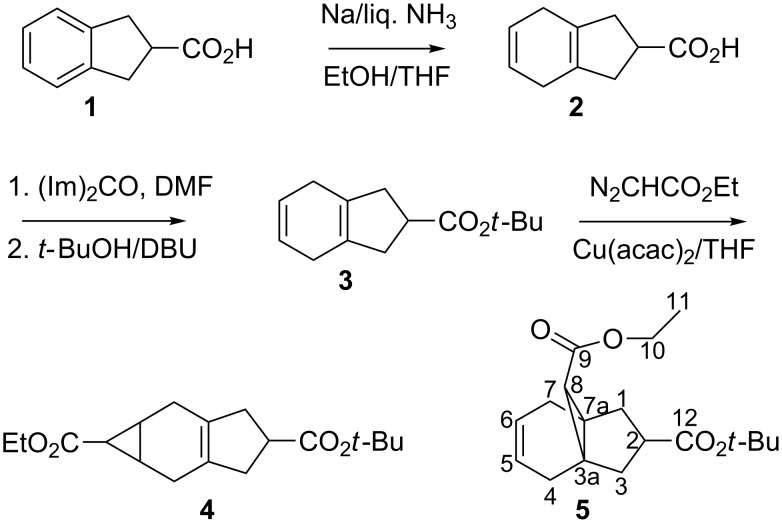
Preparation of carbene adducts **4** [18] and **5**.

The synthesis of various intermediates and the final hydroazulene derivatives **9** and **10** is described in this section. 2-Indancarboxylic acid (**1**) [14] was reduced under Birch conditions [15] to 4,7-dihydro-2-indancarboxylic acid (**2**) as the kinetically controlled product. In order to carry out carbene additions to the obtained 1,4-cyclohexadiene system, esterification [16] of **2** was carried out in a one-pot procedure in DMF at 40 °C with 1,1'-carbonyldiimidazole, *tert*-butyl alcohol and DBU to afford ester **3** in quantitative yield. The ester **3** was then subjected to carbene addition by treatment with ethyl diazoacetate in the presence of a copper catalyst [17] in THF under reflux. The carbene adduct **4** was obtained as the major product, as a colorless oil in acceptable yields (55–60%, Scheme 1). In addition to **4**, we were able to isolate the side product **5** in ca. 10% yield. The formation of carbene adduct **4** from 2-indancarboxylic acid (**1**) can also be followed from the previous work [18]. Evidently, in the formation of the adduct, the carbene attacks the central double bond of the substrate.

It is worth mentioning that in our earlier work on similar compounds [19], we isolated at least two isomers of **4** (with the methine hydrogen atom at the five-membered ring being present at either the α or β position). In our earlier work [10], carbene addition to similar compound(s) was found to give a mixture of stereoisomers. However, in the present case, we were only able to isolate **4** as a single stereoisomer. Furthermore, the procedure for the synthesis of compounds **4** and **5** is essentially the same as described previously for analogous compounds [10]. The complete characterization of intermediates **2** and **3** and carbene adduct **4** was carried out by NMR and related analytical techniques, similar to previous reports [18], while the 3D structure of **4** was further established based on the X-ray data of its derivative **6** [20].

It is important to note that the addition resulted in higher yields when the length of time used for the addition of the ethyl diazoacetate was extended, which was again similar to our earlier investigations [10]. The formation of only one major isomer (**4**) in this case could be due to the greater steric hindrance for carbene addition to the inner carbon–carbon double bond, in spite of the fact that the central olefinic double bond is more electron-rich and, hence, in principle more susceptible to such additions. In order to elucidate the configuration of **4**, ester cleavage with formic acid was carried out resulting in the acid **6** as a colorless solid, which was recrystallized from hexane and dichloromethane to afford single crystals suitable for X-ray analysis. The X-ray data [20] showed that the central, six-membered ring is almost planar but is slightly folded about the axis C(2)···C(6); the five-membered ring is essentially planar. The two larger rings are approximately coplanar, but the three- and six-membered rings form an interplanar angle of 78.4(1)°. The atoms of the ester side chain are coplanar and this plane forms a dihedral angle of 78.73(4)° with the central ring. In the crystal, the molecules form hydrogen-bonded dimers across inversion centers. Furthermore, a triplet in the ^1^H NMR spectrum at δ = 1.54 ppm with a coupling constant of 4.2 Hz between the H-atoms at the cyclopropyl ring, indicates an *anti-*relation between the ethoxycarbonyl group and the cyclohexene ring.

The subsequent step comprised the ring opening of **4** to furnish the cycloheptatrienes **7** and **8**. The carbene adduct **4** was first treated with bromine leading to the formation of the corresponding dibromide, which was then followed by elimination with triethylamine to afford triethylamine hydrobromide alongside the formation of a norcaradiene intermediate in situ, which was transformed into the 2,6-disubstituted tetrahydroazulene **7** as a viscous oil. It is important to note that tetrahydroazulenes, in contrast to perhydroazulenes, are not very resistant towards dehydrogenation (oxidation) and, when kept at room temperature for a few days, form the corresponding azulenes (giving a bluish color).

**Scheme 2:**
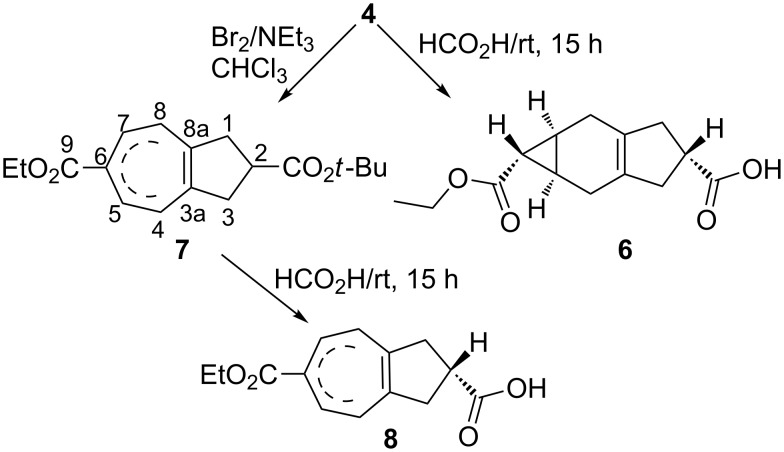
Preparation of the cycloheptatrienes **7** and **8** [18,20].

Similar to our earlier investigations [10], various isomers could be formed through the rearrangement of double bonds in the seven-membered ring of **7**. However, through the NMR data of **7**, we saw the formation of an isomer with double bonds at the ring junction as a major isomer. Formation of one major isomer under the given conditions could be explained through the hydrogen shifts of cycloheptatrienes by the so-called Berson–Willcott rearrangement [21]. We assume that bromination (Br_2_) and dehydrobromination (Et_3_N) of **4** resulted in the formation of an intermediate, which converted to **7** through thermal disrotatory electrocyclic ring opening and a base-catalyzed prototropic shift. The ester **7** underwent acid-catalyzed hydrolysis (formic acid) to afford **8** as a solid, which on recrystallization resulted in single crystals suitable for X-ray analysis (Scheme 2).

**Scheme 3:**
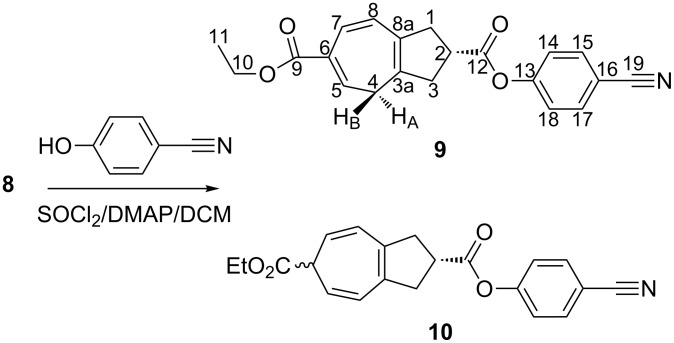
Preparation of derivatives **9** and **10**.

The structural analysis of **8** revealed [22] an azulene framework that is partially hydrogenated to a cyclopentane subunit in places where molecules are slightly disordered in the lattice. These molecules represent the slightly torsionally distorted enantiomers of **8**. At this stage we did not find any hydrogen atom at position 6; however, additional H-atoms at C(4) and C(8), each with only 50% occupancy, were found. Acid **8** was further condensed [23] with *p*-cyanophenol in the presence of SOCl_2_ and DMAP in dichloromethane to obtain the corresponding ester. The GC analysis of the product mixture indicated the presence of two isomers (ratio 3:1), which were later separated through reversed-phase HPLC (MeOH/H_2_O 3:1) and characterized as derivatives **9** and **10** (Scheme 3).

The cycloheptatriene unit of **9** adopts, in sterically relaxed systems, a boat-like conformation, whereas its planar form represents the transition state of the thermal boat-inversion process. Additionally, since **8** and **9** possess a center of chirality at C(2), there are two different boat conformations of the cycloheptatriene unit possible, namely those in which C(4) is on the same or on the opposite side with respect to the substituent at C(2). The ^1^H NMR spectrum (400 MHz, CDCl_3_) of **9** displays diastereotopic (*syn* and *anti* to the ester group) H-atoms at C(4), indicating that the ring-inversion process is still slow at the temperature of the measurement, but the observed dd signals already show some line broadening.

Structure elucidation of compounds **9** and **10** was carried out by 1D and 2D NMR spectroscopy. Most ^13^C-signals could be assigned through HMQC analysis. For quaternary carbons, analysis of HMBC data was sufficient to complete the assignment. In the ^1^H NMR spectrum of compound **9**, the H-atom (H_A_ or H_α_) at C(1) is located in the deshielding region of the carbonyl function and, hence, resonates slightly downfield (from its geminal H-atom, H_B_) at δ = 2.94 ppm, together with the H-atoms at C(3), whereas H_B_ (H_β_) itself resonates at δ *=* 2.92 ppm. Furthermore, the H-atom at C(2), being geminal to a strongly deshielding carboxylate group, gives a multiplet at δ = 3.49 ppm. Additionally, H-atoms at C(14) and C(18) show a doublet at δ = 7.22 ppm (*J* = 8.7 Hz), which is acceptable for such protons after considering the effect of cyano and carboxylate groups on the respective positions of the aromatic ring. The doublet at δ = 7.65 ppm (*J* = 8.7 Hz), integrated for two protons, was assigned to the H-atoms at C(15) and C(17). The most differentiated signals were identified for H-atoms at C(4) giving two distinct dd at δ *=* 2.50 ppm (*J* = 7.1, 13.4 Hz) and at δ = 2.65 ppm (*J* = 7.5, 13.5 Hz). These values seem very reasonable considering the envelope form of the seven-membered ring, in which C(4) extends out of the plane of the ring thereby allowing the remaining six carbon atoms to enhance the delocalization. This six-carbon system in turns generates a ring current similar to benzene, though less pronounced, allowing H_A_ to resonate in the deshielding region (δ = 2.65 ppm) and H_B_ in the shielding region (δ = 2.50 ppm). The H-atoms at positions C(5) and C(8) of the seven-membered ring (being almost in identical electronic environments), give a broad multiplet at δ = 6.52 ppm. However, a pronounced doublet at δ *=* 6.96 ppm (*J* = 11.2 Hz) is assigned for the hydrogen atom at position C(7) considering the effect of the nearby carboxylate group and several sp^2^-hybridized carbon atoms in its immediate vicinity on both sides.

In the case of compound **10**, due to shifting of the double bonds, the single H-atom at C(4) becomes olefinic (conjugated) thereby resonating together with C(8); it displays a doublet at δ *=* 5.47 ppm (*J* = 9.2 Hz). Similarly, the H-atoms at C(5) and C(7) give dd at δ = 6.22 ppm (*J* = 5.6, 9.1 Hz). The single proton at C(6) provides a triplet at δ *=* 2.73 ppm (*J* = 5.6 Hz). However, the exact stereochemistry at C(6) could not be established in the present case.

Finally, we investigated compounds **9** and **10** under a polarizing microscope for their textural features, and DSC experiments were carried out to determine the phase-transition temperatures. In the case of **9**, the first melting-point peak in the DSC never recovered, but a very broad transition at 5 °C was observed subsequently indicating the possibility of a very exothermic process on cooling to −40 °C. Furthermore, after the first melting the sample was an isotropic liquid down to 0 °C. The first melting showed complex behavior over a temperature range of 30 °C, and areas with Schlieren-like textures were present but were never recovered or reproduced. In the case of compound **10**, the same behavior was observed. As we mentioned earlier, tetrahydroazulenes, in contrast to perhydroazulenes, are not very resistant against dehydrogenation (oxidation), and when kept at room temperature for a few days, they form the corresponding azulenes. Such an unusual behavior of compounds **9** and **10** can easily be attributed to their oxidation. Additionally, our earlier investigations on similar compounds [10,13] showed that fully hydrogenated derivatives show pronounced mesogenic behavior.

## Experimental

Thin-layer chromatography was performed by using precoated plastic plates, PolyGram Sil G/UV_254_. Column chromatography was performed on silica gel 60 (70–230 mesh) from Merck (Darmstadt). HPLC analysis was carried out on a system consisting of Shimadzu (Duisburg, Germany), Gilson 305 pumps, a Shimadzu SPD-M10AV UV–vis detector, and a fraction collector. In the GC system, a Hewlett-Packard 6890 with a flame-ionization-detection (FID) system was used. All chromatograms were processed by ColaChrom software (Version 8.1) developed by MPI für Kohlenforschung, Mülheim, Germany. All organic solvents used in the study were HPLC grade and were purchased from Baker GmbH, Germany. The argon gas used was of high-purity grade. IR spectra were recorded using a Nicolet 320 FT-IR and a Bruker Tensor 27 spectrometer. Samples were prepared either as KBr pellets or as thin films. ^1^H and ^13^C NMR spectra were recorded on the following spectrometers: Bruker AC-200, ^1^H NMR (200.1 MHz), ^13^C NMR (50.3 MHz); Bruker DRX-400, ^1^H NMR (400.1 MHz), ^13^C NMR (100.6 MHz). Chemical shifts (δ) are expressed in parts per million (ppm) downfield from tetramethylsilane or by using the residual nondeuterated solvent as the internal standard (CDCl_3_: ^1^H, δ = 7.26 ppm; ^13^C, δ = 77.00 ppm). Coupling constants are expressed in hertz. Mass spectra were recorded by using a Finnigan MAT 8430 spectrometer using the electron-ionization method (EI, 70 eV). Synthesis and analytical data of compounds **2**–**4** and **6** can be found in the literature [18].

**Ethyl 2-*tert*-butoxycarbonyl-2,3,4,7-tetrahydro-1*H*-3a,7a-methanoinden-8-carboxylate (5):** Compound **5** was obtained as a highly viscous, colorless oil in 10% yield during the synthesis of carbene adduct **4** as given in the literature [18]. *R*_f_ 0.41 (SiO_2_; hexane/CH_2_Cl_2_ 1:1); IR (film): 2900 (m, CH-stretch), 1695 and 1700 (s, C=O), 1340, 1250, 1133 (m) cm^−1^; ^1^H NMR (200.1 MHz, CDCl_3_) δ 1.21 (t, *^3^J* = 7.1 Hz, H-C(11)), 1.31–1.40 (s, C(CH_3_)_3_), 1.55–1.68 (m, CH_2_(1), CH_2_(3)), 1.62 (br s, H-C(8)), 2.31 (br s, CH_2_(4), CH_2_(7)), 2.78–2.81 (m, H-C(2)), 4.08 (q, *J* = 7.1 Hz, CH_2_(10)), 5.49 ppm (br s, H-C(5), H-C(6)); ^13^C NMR (50.3 MHz, CDCl_3_) δ 14.1 (q, C(11)), 27.80 (q, C(*C*H_3_)_3_), 29.41 (d, C(8)), 31.23 (t, C(4), C(7)), 34.31 (s, C(3a), C(7a)), 38.23 (d, C(2)), 42.68 (d, C(1), C(3)), 61.01 (t, C(10)), 80.12 (s, *C*(CH_3_)_3_), 125.60 (d, C(5), C(6)), 171.84, 172.01 (s, C(9), C(12)); EIMS *m*/*z*: 306 (8, M*^+^*), 249 (57, [M − C_4_H_9_]*^+^*), 205 (80, [M − C_5_H_9_O_2_]*^+^*), 132 (100, [205 − C_3_H_5_O_2_]*^+^*); HRMS (*m*/*z*): [M]^+^ calcd for C_18_H_26_O_4_, 306.183109; found, 306.183123 + 1.1 ppm.

**6-Ethyl-tetrahydroazulene-2-carboxylate (8):** To a stirred solution of **4** (3.06 g, 0.01 mol) in CHCl_3_ (100 mL), a solution of Br_2_ (0.6 mL, 0.01 mol) in CHCl_3_ (5 mL) was added dropwise at 0 °C. When the addition was complete, triethylamine (6.91 mL, 49.71 mmol) was added. Triethylamine hydrobromide began to form immediately. The mixture was heated under reflux for 18 h. After cooling, the hydrobromide was filtered off. The filtrate was evaporated and the resulting oil partitioned between benzene and dilute aqueous acid (HCl). The benzene layer was washed with water, dried with MgSO_4_ and filtered, and the solvent was removed. The reaction mixture was filtered through a small column of silica gel, eluting with pentane and dichloromethane (1:2). After evaporation of the solvent the crude product **7** was obtained as a viscous bluish oil (2.6 g, 85%). The preparation of compound **7** is analogous to that described in [10]. The crude intermediate **7** was not purified further and was subjected to hydrolysis directly: A solution of **7** (1.0 g, 3.29 mmol) in formic acid (100 mL) was stirred at rt for 15 h. The solvent was removed under reduced pressure and the crude product was recrystallized from hexane and dichloromethane to give 0.71 g (85%) of **8** as slightly bluish needles. *R*_f_ 0.35 (SiO_2_; EtOAc/CH_2_Cl_2_ 1:5); IR (film): 2913 (m, CH, stretch.), 1712, 1718 (s, C=O), 1320, 1150 (m) cm^−1^; ^1^H NMR (200.1 MHz, CDCl_3_) δ 1.29 (t, *^3^J* = 7.1 Hz, CH_3_(12)), 2.80–3.27 (m, CH_2_(1), CH_2_(3), H-C(2)), 4.18 (q, *J* = 7.1 Hz, CH_2_(11)), 2.60–2.75/5.48–6.69 ppm (2 × m*,* 5 H, seven-membered ring-H); ^13^C NMR (50.3 MHz, CDCl_3_) δ 14.21 (q, C(12)), 27.50 (t), 37.53 (t), 39.39 (t), 41.97 (d), 60.79 (d, C(11)), 117.87 (d), 124.60 (d), 126.61 (d), 130.61 (d), 133.99 (s), 132.70 (s), 166.81, 181.41 ppm (s, C(9), C(10)); EIMS *m*/*z*: 248 (19, M*^+^*), 219 (59, [M − C_2_H_5_]*^+^*), 203 (18, [M − C_2_H_5_O]*^+^*), 175 (72, [M − C_3_H_5_O_2_]*^+^*), 129 (100, [175 − HCO_2_H]*^+^*); HRMS (*m*/*z*): [M]^+^ calcd for C_14_H_16_O_4_, 248.104859; found, 248.104835 + 0.96 ppm.

**1,2,3,4-Tetrahydroazulene-2,6-dicarboxylic acid 2-(4-cyanophenyl) ester 6-ethyl ester (9):** Thionyl chloride (0.021 mL, 0.28 mmol) was added to a solution of 4-(*N*,*N*-dimethylamino)pyridine (34 mg, 0.27 mmol) in dichloromethane (5 mL) at −20 °C. Acid **8** (70 mg, 0.28 mmol) was added and the resulting solution was stirred for 1 h. Then, 4-(*N*,*N*-dimethylamino)pyridine (34 mg, 0.27 mmol) and the *p*-cyanophenol (31 mg, 0.30 mmol) in dichloromethane were added and the stirring was continued for another 1 h. The mixture was washed with water (5 mL), and the organic layer was separated and dried with sodium sulfate to give a crude mixture of **9** and **10** (75 mg, 72%). The product mixture was filtered through a small column of silica gel, eluting with pentane and dichloromethane (1:2), to remove impurities, and was further separated through reversed-phase HPLC. The analytical HPLC indicated the presence of both isomers **9** and **10** in a 3:1 ratio. For preparative HPLC, the sample was dissolved in MeOH/CH*_2_*Cl_2_, and MeOH/H_2_O 75:25 (v/v) was used as a mobile phase. Compound **9** was isolated in 97% purity (*t*_R_ 4.40 min), while **10** was obtained in 94% purity (*t*_R_ 4.98). R_f_ 0.32 (SiO_2_; hexane/CH_2_Cl_2_ 1:1); mp 83–84 °C; IR (film): 2931 (m CH, stretch), 1705 and 1714s (C=O), 1311, 1230, 1122 (m) cm^−1^; ^1^H NMR (400.1 MHz, CDCl_3_) δ 1.29 (t, *^3^J* = 7.1 Hz, CH_3_(11)), 2.50 (dd, *J* = 7.1, 13.4 Hz, H_β_(4)), 2.65 (dd, *J* = 7.5, 13.5 Hz, H_α_(4)), 2.92 (m, CH_2_(3), H_β_(1)), 2.94 (dd, *J* = 6.8, 17.4 Hz, H_α_(1)), 3.49 (m, H-C(2)), 4.21 (q, *J* = 7.1 Hz, CH_2_(10)), 6.52 (br. m, H-C(5), H-C(8)), 6.96 (d, *J* = 11.2 Hz, H-C(7)), 7.22 (d, *J* = 8.7 Hz, H-C(14), H-C(18)), 7.65 ppm (d, *J* = 8.7 Hz, H-C(15), H-C(17)); ^13^C NMR (100.6 MHz, CDCl_3_) δ 14.01 (q, C(11)), 27.26 (t, C(4)), 37.43 (t, C(3)), 39.63 (t, C(1)), 42.01 (d, C(5)), 60.63 (t, C(10)), 109.49 (s, C(19)), 117.94 (s, C(16)), 122.39 (d, C(2)), 126.74 (d, C(8)), 128.50 (2 × d, C(15), (17)), 130.47 (d, C(7)), 130.81 (s, C(3a), 132.27 (s, C(8a)), 133.50 (2 × d, C(14), C(18)), 133.61 (s, C(6)), 153.83 (s, C(13)), 166.52 (s, C(9)), 172.76 ppm (s, C(12)); EIMS *m*/*z*: 349 (27, [M*^+^*]), 320 (100, [M − C_2_H_5_]*^+^*), 304 (17, [M − C_2_H_5_O]*^+^*), 276 (18, [M − C_3_H_5_O_2_]*^+^*), 203 (56, [M − C_8_H_4_O_2_N]*^+^*); HRMS (*m*/*z*): [M + Na]^+^ calcd for C_21_H_19_NO_4_Na, 372.120362; found, 372.120629 + 0.72 ppm.

**1,2,3,6-Tetrahydroazulene-2,6-dicarboxylic acid 2-(4-cyanophenyl) ester 6-ethyl ester (10): ***R*_f_ 0.29 (SiO_2_; hexane/CH_2_Cl_2_ 1:1); mp 80–82 °C; IR (film): 2927 (m, CH-stretch), 1703, 1712 (s, C=O), 1342, 1223, 1165 (m) cm^−1^; ^1^H NMR (400.1 MHz, CDCl_3_) δ 1.29 (t, *^3^J* = 7.1 Hz, CH_3_(11)), 2.73 (t, *J* = 5.6 Hz, H-C(6)), 3.16 (m, CH_2_(1), CH_2_(3)), 3.42 (m, H-C(2)), 4.25 (q, *J* = 7.1 Hz, CH_2_(10)), 5.47 (d, *J* = 9.2 Hz, H-C(4), H-C(8)), 6.22 (dd, *J* = 5.6, 9.1 Hz, H-C(5), H-C(7)), 7.23 (d, *J* = 8.8 Hz, H-C(15), H-C(17)), 7.69 (d, *J* = 8.8 Hz, H-C(14), H-C(18)); ^13^C NMR (100.6 MHz, CDCl_3_) δ 14.0 (q, C(11)), 39.24 (t, C(1), C(3)), 40.95 (d, C(6)), 45.0 (d, C(2)), 60.9 (t, C(10)), 109.56 (s, C(19)), 117.94 (s, C(16)), 118.08 (2 × d, C(5), C(7)), 122.39 (2 × d, C(4), C(8)), 124.28 (2 × d, C(15), C(17)), 133.45 (2 × d, C(14), C(18)), 139.44 (2 × s, C(3a), C(8a)), 153.83 (s, C(13)), 172.50 (s, C(9)), 172.80 ppm (s, C(12)); EIMS 349 (26, [M*^+^*]), 320 (100, [M − C_2_H_5_]*^+^*), 304 (16, [M − C_2_H_5_O]*^+^*), 276 (16, [M − C_3_H_5_O_2_]*^+^*), 203 (57, [M − C_8_H_4_O_2_N]*^+^*); HRMS (*m*/*z*): [M + Na]^+^ calcd for C_21_H_19_NO_4_Na, 372.120362; found, 372.120630 + 1.05 ppm.

## Supporting Information

File 1 DSC-data of isomers **9** and **10**.
