# Supplementary material for: Synthesis of 2,6-disubstituted tetrahydroazulene derivatives
Source: Beilstein J Org Chem. 2012 May 4;8:693–8. doi: 10.3762/bjoc.8.77 (PMC3388855; doi:10.3762/bjoc.8.77)
Supplement: File 1 — DSC-data of isomers 9 and 10. [file Beilstein_J_Org_Chem-08-693-s001.pdf]

Supporting Information

for

Synthesis of 2,6-disubstituted tetrahydroazulene derivatives

Zakir Hussain*[1,2], Henning Hopf[1], Khurshid Ayub[2] and S. Holger Eichhorn[3]

Address: [1]Institut für Organische Chemie, Technische Universität Braunschweig, Hagenring 30, D-38106 Braunschweig, Germany; Fax: +49 5313915388, [2]Department of Chemistry, COMSATS Institute of Information Technology (CIIT), University Road, Abbottabad 22060, KPK, Pakistan; Fax: +92 (992) 383441 and [3]Department of Chemistry and Biochemistry, University of Windsor, 401 Sunset Avenue, Essex Hall, Windsor, ON Canada N9B 3P4, Fax: +1 (519) 973-7064

Email: Zakir Hussain - chem63@yahoo.com

*Corresponding author

DSC-data of isomers **9** and **10**.

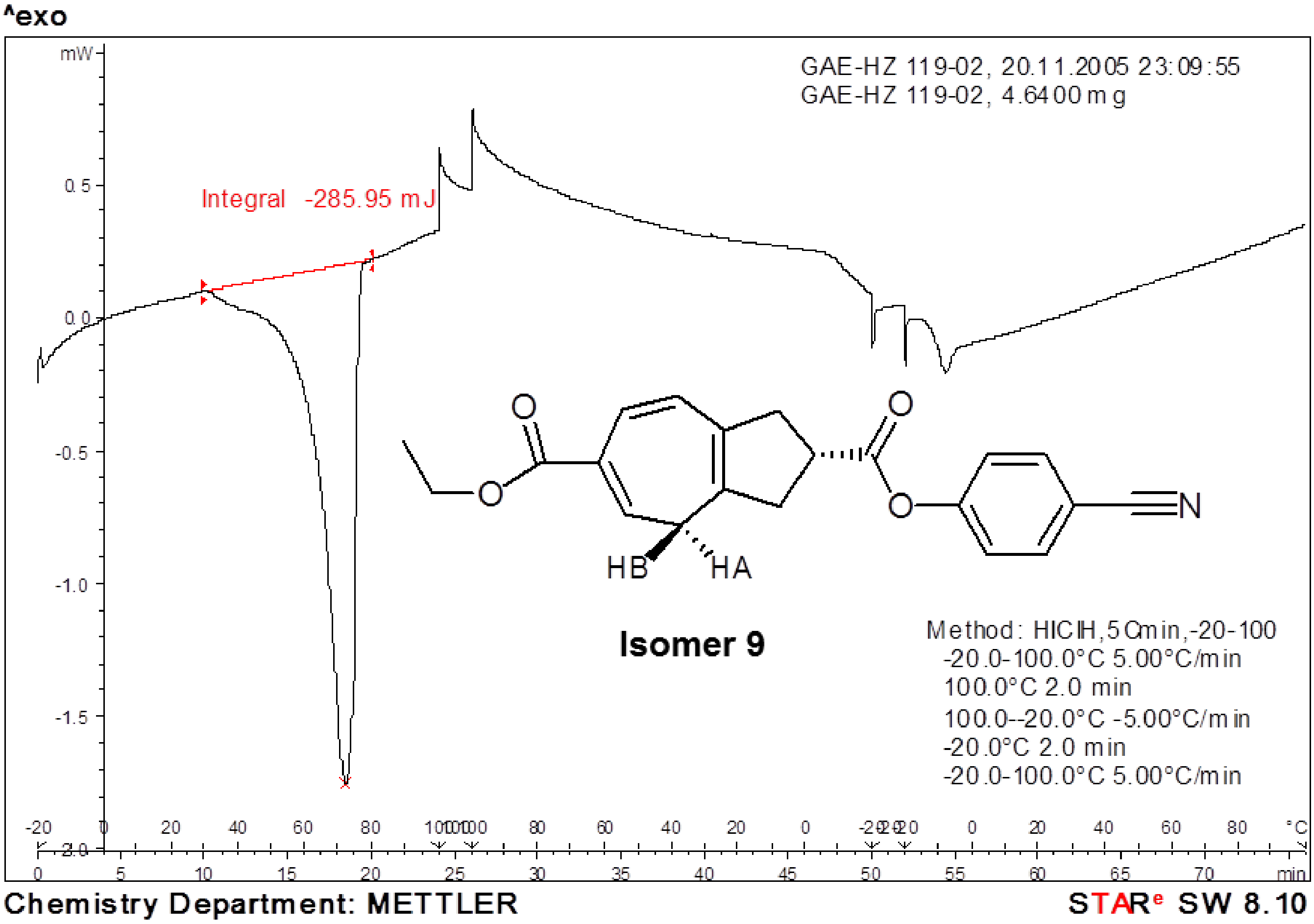
^exo
mW
GAE-HZ 119-02, 20.11.2005 23:09:55
GAE-HZ 119-02, 4.6400 mg
Integral -285.95 mJ
HB
HA
Isomer 9
Method: HIClH,5Cmin,-20-100
-20.0-100.0°C 5.00°C/min
100.0°C 2.0 min
100.0--20.0°C -5.00°C/min
-20.0°C 2.0 min
-20.0-100.0°C 5.00°C/min
°C
min
Chemistry Department: METTLER
STARe SW 8.10

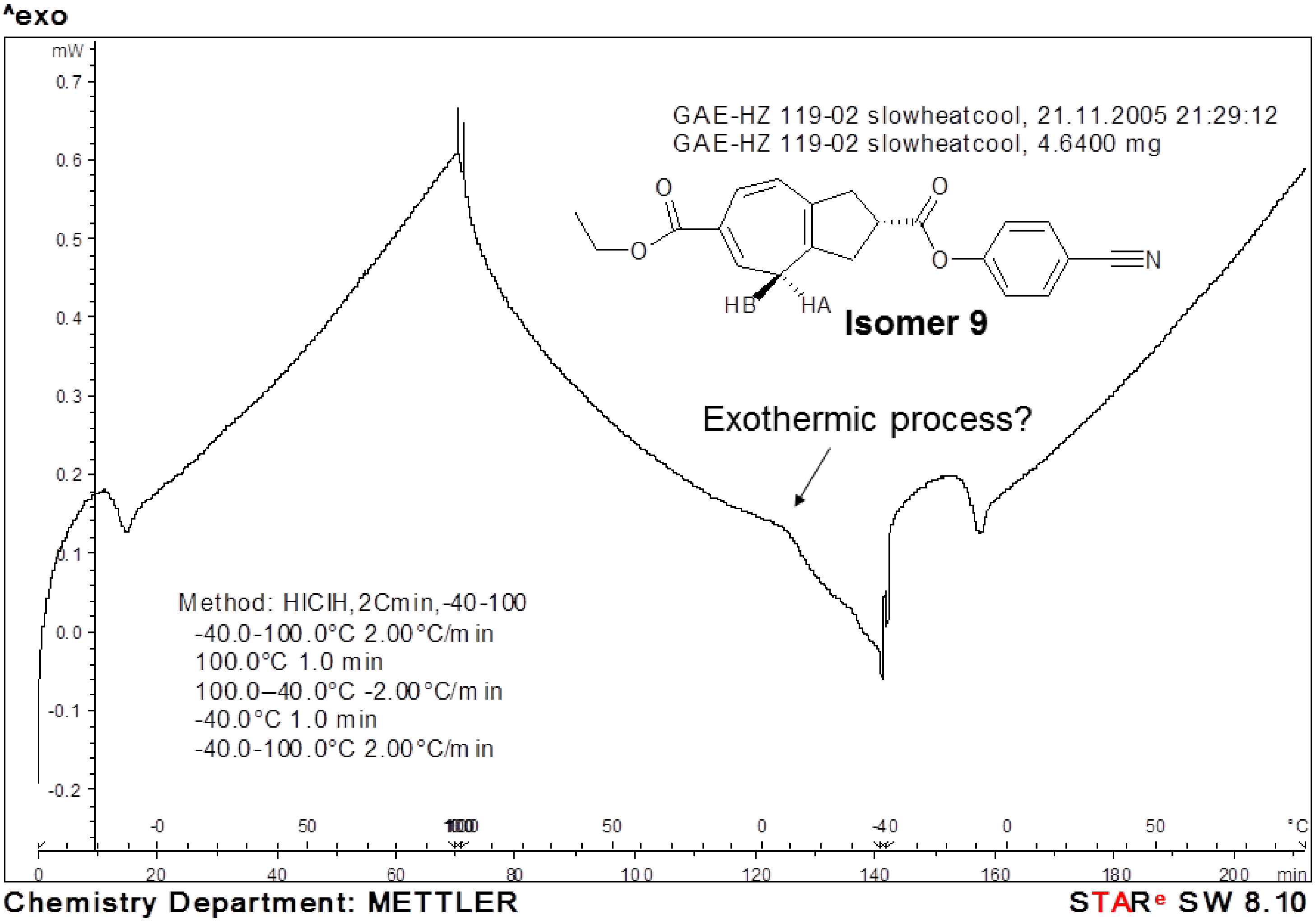
^exo
mW
GAE-HZ 119-02 slowheatcool, 21.11.2005 21:29:12
GAE-HZ 119-02 slowheatcool, 4.6400 mg
HB
HA
Isomer 9
Exothermic process?
Method: HICIH,2Cmin,-40-100
-40.0-100.0°C 2.00°C/min
100.0°C 1.0 min
100.0–40.0°C -2.00°C/min
-40.0°C 1.0 min
-40.0-100.0°C 2.00°C/min
°C
min
Chemistry Department: METTLER
STARe SW 8.10

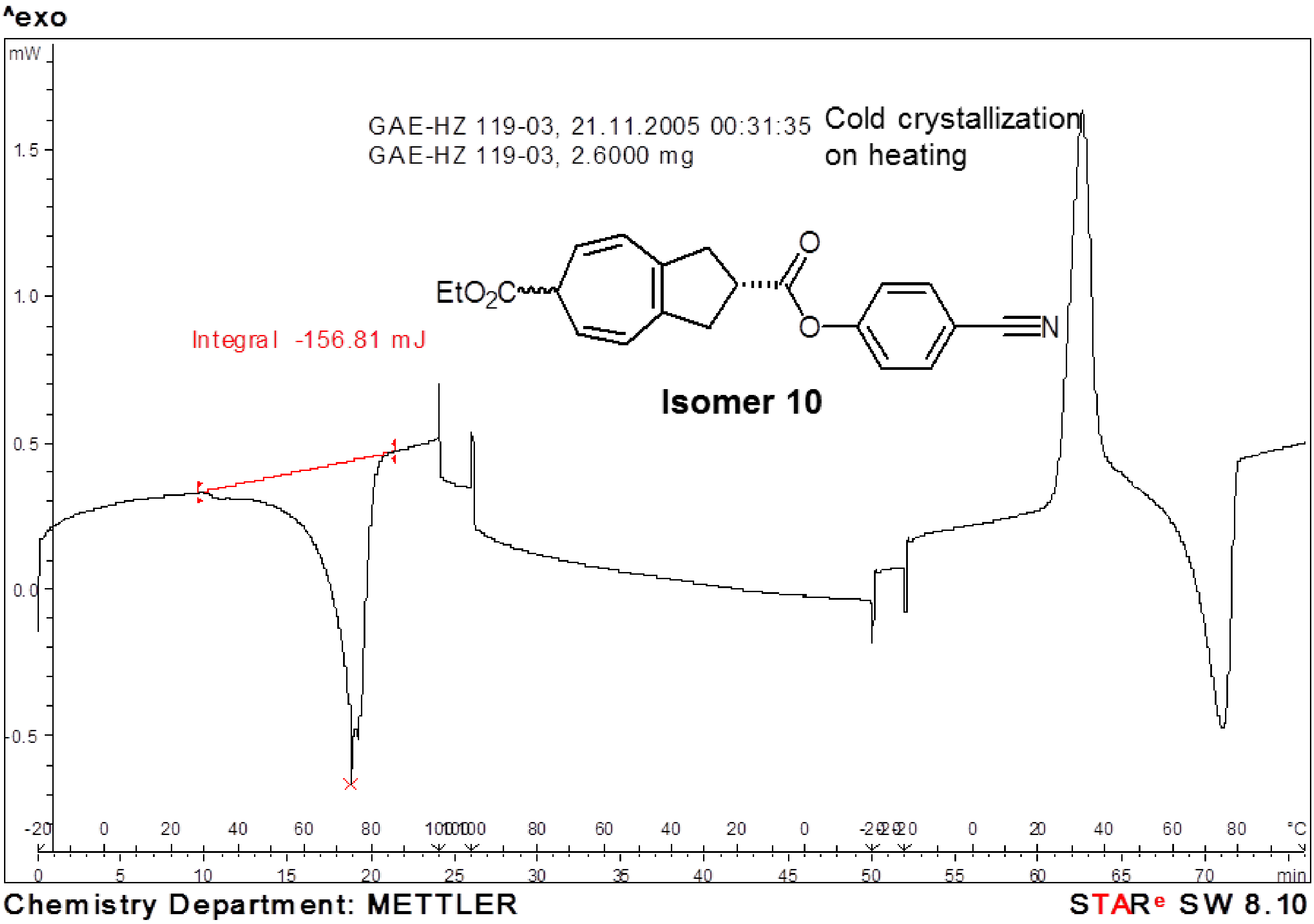
^exo
mW
GAE-HZ 119-03, 21.11.2005 00:31:35
GAE-HZ 119-03, 2.6000 mg
Cold crystallization on heating
Integral -156.81 mJ
EtO2C
O
O
N
Isomer 10
°C
min
Chemistry Department: METTLER
STARe SW 8.10

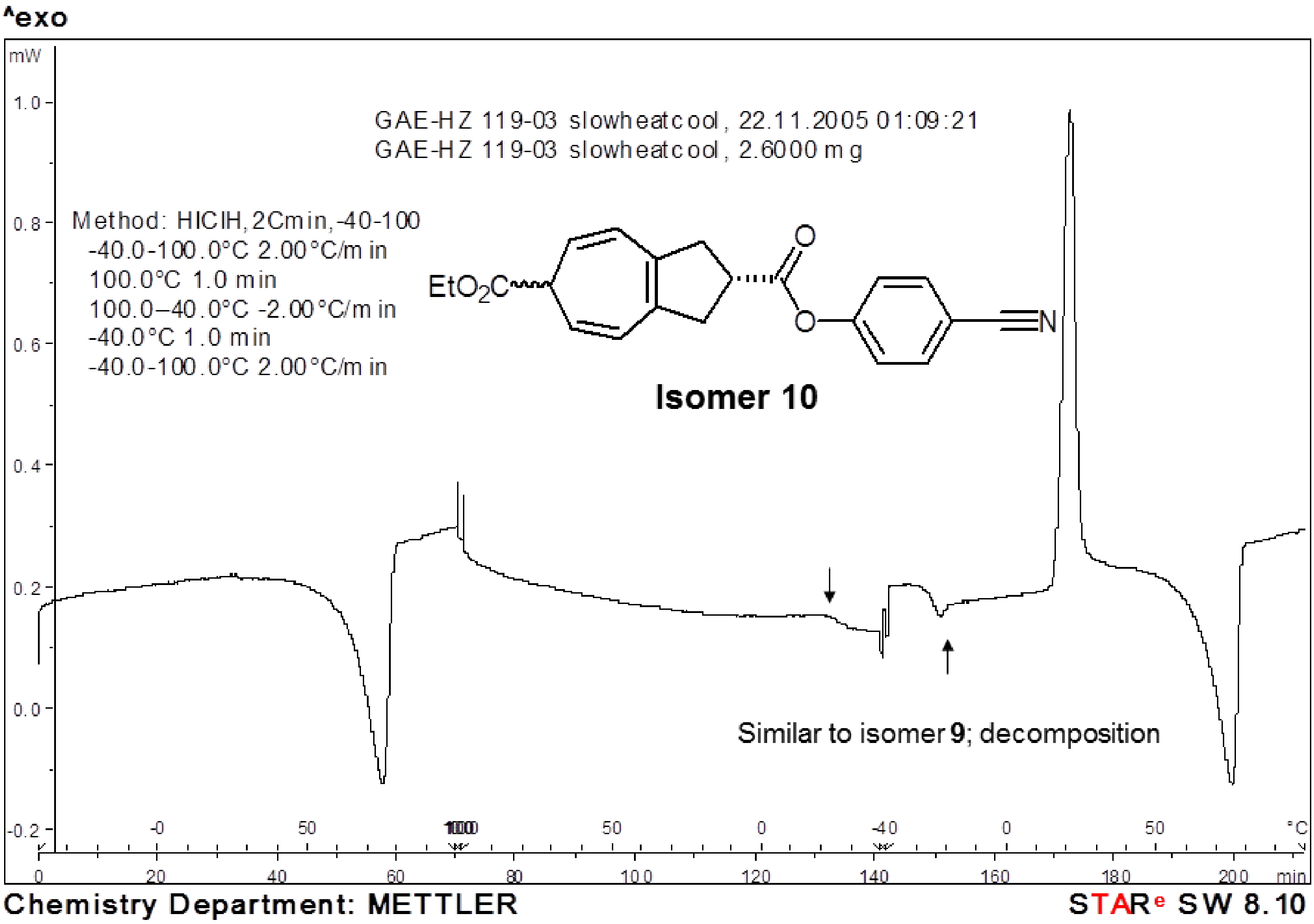
^exo
mW
GAE-HZ 119-03 slowheatcool, 22.11.2005 01:09:21
GAE-HZ 119-03 slowheatcool, 2.6000 mg
Method: HICIH,2Cmin,-40-100
-40.0-100.0°C 2.00°C/min
100.0°C 1.0 min
100.0–40.0°C -2.00°C/min
-40.0°C 1.0 min
-40.0-100.0°C 2.00°C/min
EtO2C
O
O
N
Isomer 10
Similar to isomer 9; decomposition
°C
min
Chemistry Department: METTLER
STARe SW 8.10
